# Paget Disease of the Vulva: Diagnosis by Immunohistochemistry

**DOI:** 10.1155/2015/162483

**Published:** 2015-04-28

**Authors:** Andressa Gonçalves Amorim, Brunelle Batista Fraga Mendes, Rodrigo Neves Ferreira, Antônio Chambô Filho

**Affiliations:** ^1^Department of Obstetrics and Gynecology, Santa Casa de Misericórdia Hospital, 29025-023 Vitória, ES, Brazil; ^2^Pathology Department, Santa Casa de Misericórdia Hospital, Dr. João dos Santos Neves Street 143, 29025-023 Vitória, ES, Brazil

## Abstract

The objective of this paper is to report a case of extramammary Paget disease of the vulva, to describe its diagnosis, surgical treatment, and outcome, and to discuss the general characteristics of this pathology. This is a rare neoplasm, found principally in areas in which apocrine and eccrine glands are numerous. This case report is relevant to the literature since the differential diagnosis of extramammary Paget disease is difficult to be done only with the macroscopic appearance of the lesion and even with the microscopic characteristics, requiring further studies, immunohistochemistry, as to differentiate pathologies. The present report describes the case of a 63-year-old patient at the Santa Casa de Misericórdia Hospital in Vitória, Espírito Santo, Brazil, who presented with a hardened, ulcerated, and purplish lesion with hyperchromic and hypochromic spots, measuring 4 cm in diameter, located on the lower third of right labium majus, close to the vaginal fourchette. A right hemivulvectomy was performed, leaving wide margins all around. The patient progressed satisfactorily following surgery. Although extramammary Paget disease is rare, its incidence increases as a function of the patient's age. Patients should be followed up closely because of the risk of persistence and/or recurrence of the disease.

## 1. Introduction

James Paget was the first to describe Paget disease (PD) in 1874, while extramammary Paget disease (EMPD) was first described by Crocker in 1888 [1]. The condition consists of an intraepithelial adenocarcinoma.

In general, EMPD lesions are found in areas such as the vulva, anus, perianal region, and axillae in which the density of apocrine glands is high. In women, the most common site of EMPD is the vulva; however, EMPD is responsible for less than 1% of all vulvar neoplasms [2]. Diagnosis of EMPD usually occurs between 50 and 80 years of age, with the disease being more common in Caucasian women [3]. The most common clinical symptom is pruritus. The lesion may be erythematous or eczematous, with islands of hyperkeratosis [4].

Surgical resection with wide margins is considered the standard treatment; however, successful surgical excision of the disease is a challenge and recurrences are common. Alternative treatments such as photodynamic therapy, laser therapy, radiotherapy, topical treatments such as 5% imiquimod cream, or even chemotherapy have been the subjects of debate and it is important to evaluate the available evidence [5].

The present paper reports a case with histopathological findings of extramammary Paget disease in a patient receiving care at the Department of Obstetrics and Gynecology, Santa Casa de Misericórdia Hospital, Vitória, Espírito Santo, Brazil. Prior to publication, the paper was submitted to and approved by the internal review board, reference number 35177714.70000.5065. The patient gave her written consent for the publication of this report and the accompanying images.

## 2. Case Presentation

The patient in question is a 63-year-old, married, black female patient, who became menopausal at 48 years of age. She had controlled hypertension, was not in use of hormone therapy, had no family history of gynecological cancer, and reported being a nonsmoker. She was seen at the Gynecology Department's Vulva Clinic with a complaint of intense vulvar pruritus over a prolonged period of time, associated with the appearance of a purplish lesion with white striae three months previously. She reported no other signs or symptoms.

**Figure 1:**
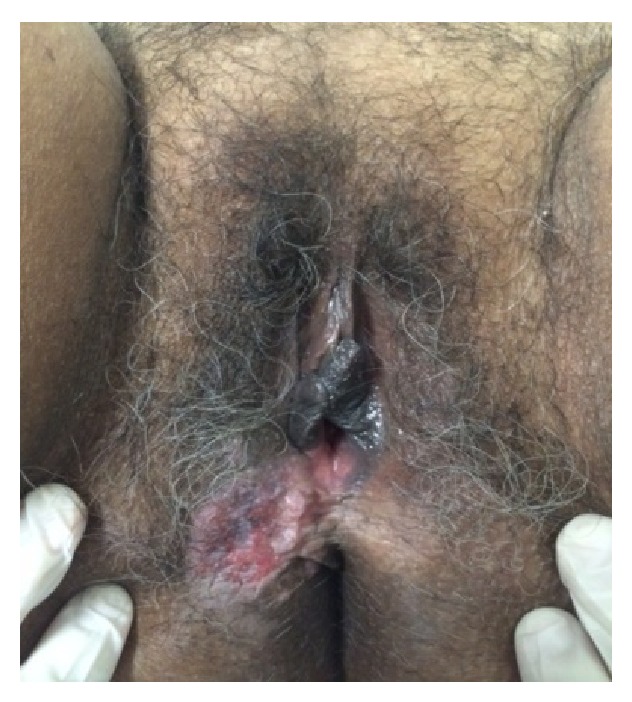
Photograph of the lesion prior to surgery.

Physical examination revealed that the patient was in a good general state of health, well hydrated, and afebrile, with no signs of anemia, jaundice, or cyanosis. Physical examination of the cardiovascular and respiratory systems and of the abdomen showed no abnormalities. Breasts were voluminous and pendulous, with no nodulations or retractions. Examination of the vulva revealed normal hair distribution for age, a hypertrophic vulva, and the presence of a hardened, ulcerated, purplish lesion measuring 4 cm in diameter, with hyperchromic and hypochromic spots, situated on the lower third of the right labium majus, close to the vaginal fourchette (Figure 1). Speculum examination revealed a cervix with an apparently normal epithelium and external os with no pathological discharge. Manual examination revealed a closed, mobile, and painless cervix with fibroelastic consistency. Bimanual palpation showed a pelvic uterus. The inguinal lymph nodes were nonpalpable.

Colposcopy and cytology were performed, revealing an intense inflammatory process and no neoplastic cells. No abnormalities were found at bilateral mammography or on transvaginal or transabdominal ultrasonography.

An incisional biopsy was proposed. Histopathology revealed malignant intraepidermal neoplasia with large cells, karyomegaly, visible nucleolus, and clear cytoplasm. The cells were focally positive for periodic acid Schiff (PAS) staining. Blackened pigment was present in some cells from the basal layer and there was a focus of ulceration. Histopathology findings were compatible with malignant intraepithelial neoplasia. An immunohistochemical study was then carried out to differentiate between melanoma and extramammary Paget disease.

### 2.1. Immunohistochemistry

Immunohistochemistry revealed positivity for cytokeratins (CK7 and CK8/18) and negativity for S100 protein and Melan A. Taken together with the morphological features, these findings permitted a diagnosis of Paget disease to be made. Consequently, surgery (right hemivulvectomy) was proposed to remove the lesion.

### 2.2. Surgery

**Figure 2:**
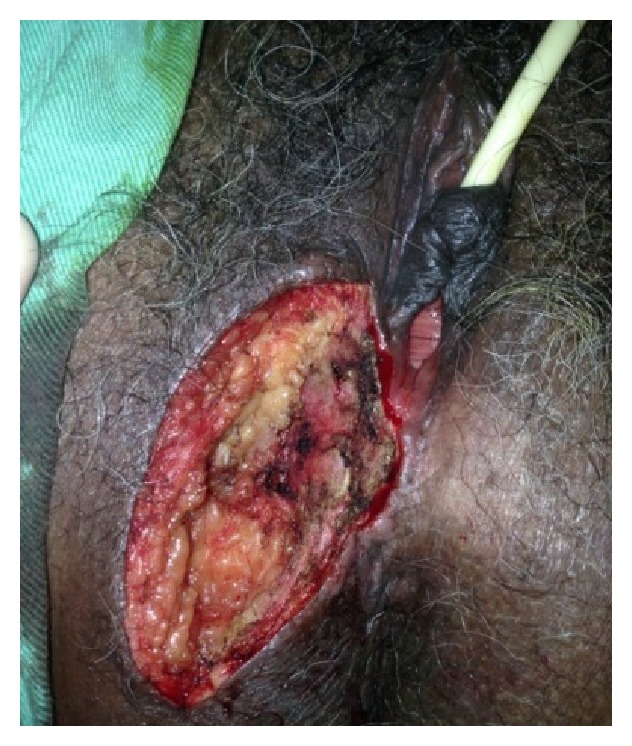
Photograph taken during surgery showing resection margins.

**Figure 3:**
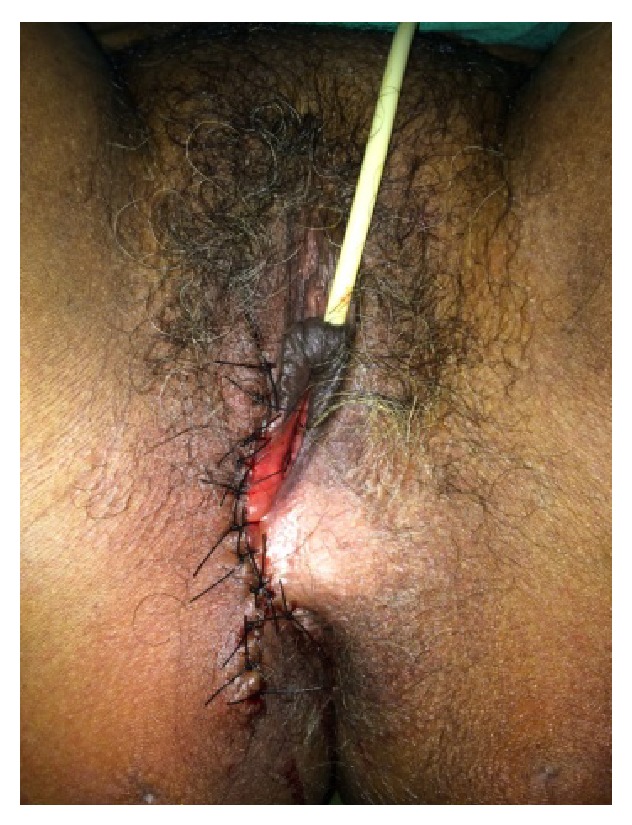
View of the completed surgical procedure.

Right hemivulvectomy was performed with wide margins all around (Figures 2 and 3). Following surgery, the patient progressed satisfactorily with no complications and was discharged from hospital.

#### 2.2.1. Histopathology of the Surgical Specimen

*Macroscopy*. There was elliptical segment of light brown, wrinkled skin measuring 5.7 × 4.5 × 3.0 cm, with a hypochromic area measuring 3.5 × 2.2 cm. Sectioned tissue appeared yellowish and elastic.

**Figure 4:**
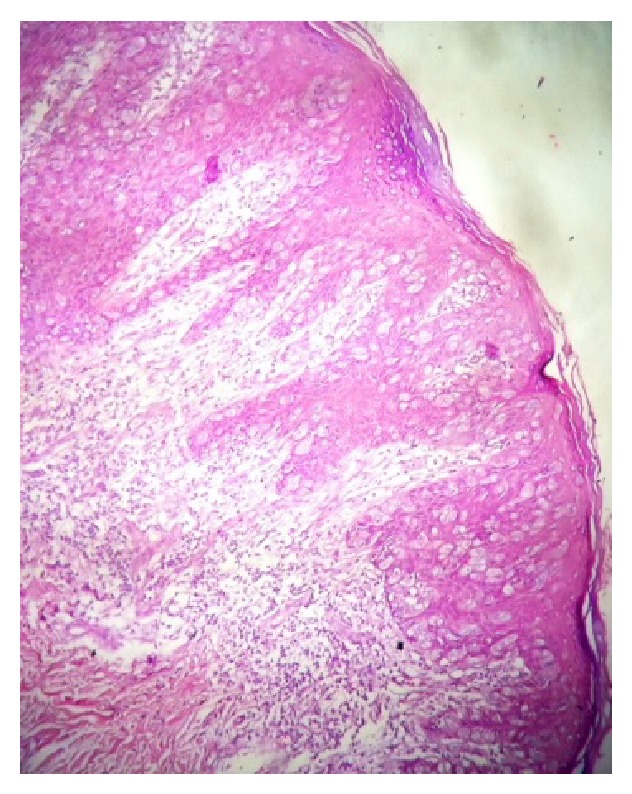
HE, 100x, Paget cells with clear cytoplasm inside the epidermis.

**Figure 5:**
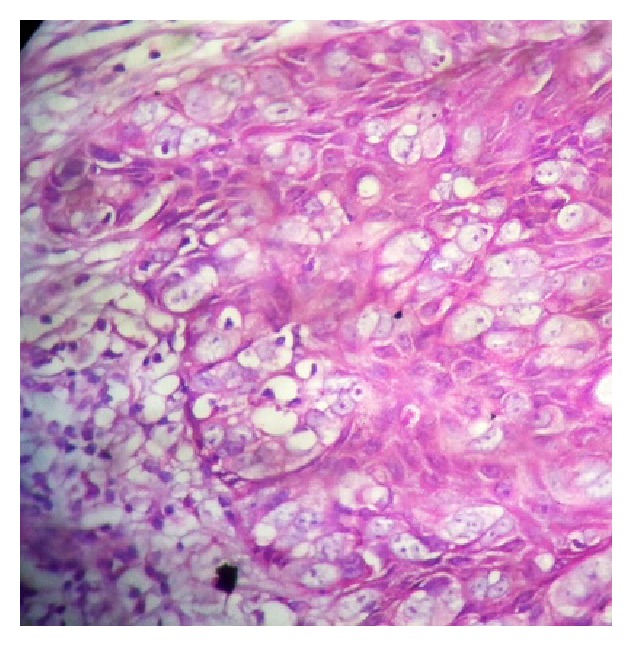
HE, 400x, Paget cells in the basal layer, some with melanic pigment.

*Microscopy*. Presence of vacuolized cytoplasm in the epidermis was detected. These cells were surrounded by a clear halo and had granular cytoplasm containing mucopolysaccharides. The nucleus was very large with a visible nucleolus. There were foci of blackened pigment compatible with melanin in the basal cells. The dermis was unaffected by the lesion. The surgical margins were free (Figures 4 and 5).

*Immunohistochemistry*.  Table 1 shows the differences in the immunohistochemical profile commonly found in skin melanomas and in Paget disease as compared to the findings in the present case, thus confirming the diagnosis.

The patient was referred to the Vulva Clinic of the Gynecology Department, Santa Casa de Misericórdia Hospital in Vitória, for follow-up.

## 3. Discussion

Extramammary Paget disease consists of a rare intraepithelial adenocarcinoma that has been described as an apocrine gland tumor. It may be benign or malignant with the potential to metastasize [6]. Nothing at all from the patient's family, social, or environmental history suggests the etiology of the development of EMPD or any predisposition towards the disease [7]. The symptoms are nonspecific. Clinical presentation is generally characterized by vulvar and perianal pruritus [8].

The clinical appearance is of an erythematous plaque with squamous or crusted areas. The size of the plaque may vary from less than 1 cm to lesions taking up the entire anogenital region. The margins of the affected region are generally clearly outlined, raised, and erythematous, resembling contact dermatitis, eczema, or even a bacterial infection. The symptoms may have been present for a long period of time. There is generally a history of various unsuccessful attempts at treating the lesion dermatologically [9].

The presence of these characteristics should raise several different suspicions, and therefore a biopsy is recommended to enable a differential diagnosis to be made with other dermatological/oncological pathologies such as melanoma, squamous cell carcinoma, hidradenitis suppurativa, psoriasis, fungal infections, contact dermatitis, and lichen sclerosus, among others [8].

Paget disease is characterized microscopically by the presence of atypical cells in the epidermis, and these cells have a large nucleus, visible nucleolus, and vacuolized cytoplasm. With their high mucin content, Paget cells stain positively for acid and neutral mucopolysaccharides, with periodic acid Schiff (PAS) stain being commonly used to clarify diagnosis. In some cases, the cells may contain granules of melanin [10]. There are theories to explain the presence of this pigment in Paget disease cells: (1) production of chemotactic factor by the neoplastic cells, generating a proliferation of dendritic melanocytes, and (2) Paget cells that may phagocyte the melanin from the melanocytes. Nevertheless, the actual physiopathology remains unknown [11].

Although finding melanin in Paget disease is rare, this fact may potentially serve as a trap when trying to reach a diagnosis, since it may mimic melanoma both at clinical examination and histologically. One architectural characteristic is that in EMPD the atypical cells with pigments of melanin are situated in the suprabasal layer, whereas, in melanomas, the malignant cells usually also surround the dermoepidermal junction [12].

If necessary, diagnosis can be confirmed using immunohistochemistry. In Paget disease, the positive epithelial markers are CK7, EMA, CEA, and mucin, whereas Melan A, HMB45, and S100 are negative in Paget disease but positive in cases of melanoma. Paget cells express HER2/neu receptors and c-erb-2 oncogene, indicating a biological origin similar to that of breast carcinoma [13].

Surgical resection is the standard treatment for EMPD and most often involves local resection and reconstruction by various means [14]. Nevertheless, because of the multifocal nature of EMPD, surgery is limited as far as prognosis is concerned and is sometimes associated with severe morbidity and functional disability. Therefore, radiotherapy has been used in certain circumstances such as in elderly patients who are clinically incapacitated for surgery or as an alternative treatment in the case of patients with a recurrence of EMPD after various surgical attempts and in those who refuse to undergo surgery [15].

In one-third of cases, there may be a recurrence of EMPD irrespective of the surgical margins. The disease may also recur in skin grafts removed from another part of the body and for as long as fifteen years after treatment as a consequence of the retrograde dissemination of Paget cells through the lymph vessels from a previously occult site of metastasis. The lesion that appears as a recidivist is almost always in situ. When affecting the perianal region, the five-year recurrence rate of Paget disease is 61% [2]. Since Paget disease does not regress spontaneously and is progressive in nature, constant follow-up is required to ensure early diagnosis of recurrences. In view of the limited experience with this disease, further studies are necessary to enable a consensus to be reached in relation to the optimal treatment for EMPD.

## 4. Conclusion

EMPD is rare; however, its incidence increases as a function of the patient's age. Clinical diagnosis is difficult, since the characteristics of the disease are nonspecific and variable; therefore, histopathology and immunohistochemical studies are required to enable a differential diagnosis to be made with melanoma, hidradenitis suppurativa, psoriasis, and contact dermatitis, among other conditions. Patients should be followed up closely because of the risk of persistence and/or recurrence of the disease. This is an original case report of interest to gynecology.

## Figures and Tables

**Table 1:** Differentiation between the immunohistochemical profile commonly found in skin melanomas and that found in Paget disease in comparison to the present case.

Antigen investigated	Melanoma in situ	Paget disease	Present case
Melan A	+	−	−
S100 protein	+	−	−
Cytokeratins	−	+	+
Carcinoembryonic antigen (CEA)	−	+	+
Epithelial membrane antigen (EMA)	−	+	+

## References

[1] Barmon D., Imchen L., Kataki A., Sharma J. (2012). Extra mammary Paget′s disease of the vulva. *Journal of Mid-life Health*.

[2] Trindade E. S., Polcheira P. A., Basílio D. B., Rocha Z. N., Rocha Júnior J. L., Primo G. R. (2004). Invasive Paget’s disease of the vulva and perianal region: a case report. *Revista Brasileira de Ginecologia e Obstetrícia*.

[3] Onaiwu C. O., Ramirez P. T., Kamat A., Pagliaro L. C., Euscher E. E., Schmeler K. M. (2014). Invasive extramammary Paget's disease of the bladder diagnosed 18 years after noninvasive extramammary Paget's disease of the vulva. *Gynecologic Oncology Case Reports*.

[4] Simon S. K., Bolanča I. K., Šentija K., Kukura V., Valetić J., Škrtić A. (2010). Vulvar Paget's disease a case report. *Collegium Antropologicum*.

[5] Edey K. A., Allan E., Murdoch J. B., Cooper S., Bryant A. (2013). Interventions for the treatment of Paget's disease of the vulva. *The Cochrane Database of Systematic Reviews*.

[6] Merot Y., Mazoujian G., Pinkus G., Momtaz-T K., Murphy G. F. (1985). Extramammary Paget's disease of the perianal and perineal regions: evidence of apocrine derivation. *Archives of Dermatology*.

[7] Helm C. W., Luesley D. M. (2000). Rare tumours of the vulva. *Cancer and Pre-Cancer of the Vulva*.

[8] Márquez-Acosta G., Olaya-Guzmán EJ., Jiménez-López J., Gómez-Pue D., Pérez-Quintanilla M. (2013). Extensive Paget's disease of the vulva: case report and a conservative management proposal. *Perinatología y Reproducción Humana*.

[9] Mann J., Lavaf A., Tejwani A., Ross P., Ashamalla H. (2012). Perianal Paget disease treated definitively with radiotherapy. *Current Oncology*.

[10] Amin R. (1999). Perianal Paget's disease. *British Journal of Radiology*.

[11] Gabbi T. V. B., Valente N. Y. S., Castro L. G. M. (2006). Pigmented Paget's disease of the nipple mimicking cutaneous melanoma: importance of the immunohistochemical profile to differentiate between these diseases. *Anais Brasileiros de Dermatologia*.

[12] Vincent J., Taube J. M. (2011). Pigmented extramammary Paget disease of the abdomen: a potential mimicker of melanoma. *Dermatology Online Journal*.

[13] Vani B. R., Thejaswini M. U., Srinivasamurthy V., Rao M. S. (2013). Pigmented Paget's disease of nipple: a diagnostic challenge on cytology. *Journal of Cytology*.

[14] Berman B., Spencer J., Villa A., Poochareon V., Elgart G. (2003). Successful treatment of extramammary Paget's disease of the scrotum with imiquimod 5% cream. *Clinical and Experimental Dermatology, Supplement*.

[15] Son S. H., Lee J. S., Kim Y. S. (2005). The role of radiation therapy for the extramammary paget's disease of the vulva; experience of 3 cases. *Cancer Research and Treatment*.

